# Clinical and Genetic Evaluation of a Cohort of Pediatric Patients with Severe Inherited Retinal Dystrophies

**DOI:** 10.3390/genes8100280

**Published:** 2017-10-20

**Authors:** Valentina Di Iorio, Marianthi Karali, Raffaella Brunetti-Pierri, Mariaelena Filippelli, Giuseppina Di Fruscio, Mariateresa Pizzo, Margherita Mutarelli, Vincenzo Nigro, Francesco Testa, Sandro Banfi, Francesca Simonelli

**Affiliations:** 1 Eye Clinic, Multidisciplinary Department of Medical, Surgical and Dental Sciences, Università degli Studi della Campania *Luigi Vanvitelli*, via Pansini 5, Naples 80131, Italy; valedior@inwind.it (V.D.I.); r.brunettipierri@gmail.com (R.B.-P.); oftelena@gmail.com (M.F.); testa.francesco@gmail.com (F.T.); 2 Medical Genetics, Department of Biochemistry, Biophysics and General Pathology, Università degli Studi della Campania *Luigi Vanvitelli*, via Luigi De Crecchio 7, Naples 80138, Italy; karali@tigem.it (M.K.); giusy.difruscio@gmail.com (G.D.F.); vinnigro@gmail.com (V.N.); 3 Telethon Institute of Genetics and Medicine, via Campi Flegrei 34, Pozzuoli 80078, Italy; pizzo@tigem.it (M.P.); mutarelli@tigem.it (M.M.)

**Keywords:** inherited retinal dystrophies, early onset, next generation sequencing, genotype-phenotype correlation, retinitis pigmentosa, Leber congenital amaurosis, achromatopsia, ellipsoid zone

## Abstract

We performed a clinical and genetic characterization of a pediatric cohort of patients with inherited retinal dystrophy (IRD) to identify the most suitable cases for gene therapy. The cohort comprised 43 patients, aged between 2 and 18 years, with severe isolated IRD at the time of presentation. The ophthalmological characterization also included assessment of the photoreceptor layer integrity in the macular region (ellipsoid zone (EZ) band). In parallel, we carried out a targeted, next-generation sequencing (NGS)-based analysis using a panel that covers over 150 genes with either an established or a candidate role in IRD pathogenesis. Based on the ophthalmological assessment, the cohort was composed of 24 Leber congenital amaurosis, 14 early onset retinitis pigmentosa, and 5 achromatopsia patients. We identified causative mutations in 58.1% of the cases. We also found novel genotype-phenotype correlations in patients harboring mutations in the *CEP290* and *CNGB3* genes. The EZ band was detectable in 40% of the analyzed cases, also in patients with genotypes usually associated with severe clinical manifestations. This study provides the first detailed clinical-genetic assessment of severe IRDs with infantile onset and lays the foundation of a standardized protocol for the selection of patients that are more likely to benefit from gene replacement therapeutic approaches.

## 1. Introduction

Inherited retinal dystrophies (IRD) are a clinically and genetically heterogeneous group of disorders that affect the retina, and mainly photoreceptor cells. They represent the most frequent cause of blindness of genetic origin in the Western population [1]. To date, the clinical classification of IRDs relies on a variety of features, i.e., (a) primary target cell (cones vs. rods); (b) primarily targeted retinal region (macula vs. periphery); (c) age of onset and severity of visual dysfunction; and (d) absence or presence of extra-retinal clinical involvement (isolated vs. syndromic forms). As a result, IRDs are traditionally divided into retinitis pigmentosa (RP), achromatopsia (ACHM), and cone or cone-rod dystrophies. Among the syndromic forms, the Usher and Bardet–Biedl syndromes are the most frequent ones.

IRDs are characterized by a notable extent of genetic heterogeneity with approximately 200 responsible genes identified to date (http://www.sph.uth.tmc.edu/RetNet/). Such heterogeneity renders molecular diagnosis challenging. Next-generation sequencing (NGS) approaches, both targeted and whole exome-based [2], offer an effective solution to the problems related to the molecular characterization of highly genetically heterogeneous disorders, including IRDs. NGS-based approaches have been successfully applied to the study of the genetic basis of patients with specific forms of photoreceptor degenerations, such as RP, cone dystrophy, and Leber congenital amaurosis (LCA) [3]. Nevertheless, it is currently possible to determine the molecular defect underlying IRDs in about 50–70% of patients [4,5,6], which suggests the existence of additional genes responsible for these conditions.

A significant number of IRD cases are characterized by infantile/juvenile onset, i.e., prior to 18 years of age. This is the case of LCA, early onset retinitis pigmentosa (EORP), and ACHM. However, to the best of our knowledge, there are no reports that describe clinical phenotypes related to genotypes in a cohort of IRD patients with infantile/juvenile onset. The absence of similar reports could be justified by the many difficulties hindering a precise classification of the specific disease type in pediatric patients. Fundamental tests for a proper clinical diagnosis, such as electroretinogram (ERG), visual field (VF), or optical coherence tomography (OCT), cannot be easily carried out in these patients. Furthermore, clinical features are often shaded in young patients.

Currently, there is no effective cure for IRDs. The experimental treatment that provided initial evidence of success and currently holds higher promises is gene therapy [7,8,9]. Gene therapy is particularly relevant for those forms in which the retina retains a significant degree of morphological and functional preservation [7,8,9]. Therefore, a detailed phenotypic and genotypic characterization of pediatric IRD patients is essential because the latter represent an important reservoir of cases with high potential of successful outcomes in gene therapy-based trials. To obtain information on both genetic and clinical features of early-onset IRD cases, we analyzed a cohort of Italian IRD patients of pediatric age. We performed NGS-based genetic testing and an extensive clinical evaluation including analysis of the macular thickness (MT) and the ellipsoid zone (EZ) band [10] by OCT. We believe that the results of our study will pave the way towards the definition of a standardized pipeline to select IRD patients with the highest potential of successful outcomes in gene therapy-based approaches.

## 2. Materials and Methods

### 2.1. Ophthalmological Analysis

A total of 43 Italian patients aged between 2 and 18 years, with isolated non-syndromic retinal degeneration at the time of presentation, were recruited for the study. The medical records of the patients were taken at the Referral Center for Inherited Retinopathies of the Eye Clinic of the Università degli Studi della Campania *Luigi Vanvitelli* from June 2013 to March 2015. Inclusion criteria were: disease onset ≤10 years of age (age of symptoms’ onset), age ≤ 18 years, best corrected visual acuity (BCVA) ≤20/70, standard electroretinogram (ERG) abnormalities and macular thickness (MT) ≥100 μm in cases where ERG and OCT were performable.

Prior to enrollment in the study, all patients underwent full ophthalmological examination, which included: BCVA measured using LEA symbols in patients aged between 3–5 years or Snellen chart in patients >5 years old, Farnsworth D-15 color test, slit lamp anterior segment examination, fundus examination, Goldmann visual field examination, ERG and OCT. ERG was recorded according to the International Guidelines of the International Society of Clinical Electrophysiology of Vision (ISCEV) [11]. OCT was performed with the spectral domain OCT (SD-OCT) (Cirrus HD-OCT, Carl Zeiss, Dublin, CA, USA) by an experienced operator. The acquisition protocol comprised both a five-line raster scan and a macular cube scan pattern (512 × 128 pixels) in which a 6 × 6 mm region of the retina was scanned within a scan time of 2.4 s.

### 2.2. Clinical Diagnosis

Clinical diagnosis was performed according to the following criteria. Diagnostic criteria of LCA, according to Chacon-Camacho and Zenteno [12], included: (a) Functional signs: nystagmus already present at 6 weeks of life, photophobia, night blindness, oculodigital signs, sluggish or nearly absent pupillary responses, severe visual loss at birth; (b) Fundus appearance ranging from normal or mild retinal involvement (like “salt and pepper” dystrophy) to maculopathy or macular coloboma, bone-spicule pigment migration, marbleized fundus; (c) ERG: severely subnormal or non-detectable scotopic and photopic responses; (d) Age at diagnosis: at birth or shortly after birth [12].

Diagnostic criteria of EORP, according to Hamel [13], included: (a) Functional signs: night blindness, photophobia with visual acuity preserved in early and mid-stages; (b) Fundus appearance ranging from “salt and pepper” dystrophy to pigmentary deposits resembling bone spicules, initially in peripheral retina, attenuation of the retinal vessels, waxy pallor of the optic disc, and various degrees of retinal atrophy; (c) VF: patchy losses of peripheral vision evolving to ring shape scotoma, and eventually tunnel vision; (d) ERG: dramatic decrease in a- and b-wave amplitudes; ERG is usually unrecordable in scotopic conditions and photopic responses (30-Hz flickers, bright light) are markedly hypovolted; (e) Age: EORP is diagnosed when symptoms of mid-stage RP are already present at the age of two years [13].

Diagnostic criteria of ACHM, according Kohl et al [14], included: (a) Functional signs: nystagmus, photophobia, reduced or complete loss of color discrimination, reduced visual acuity, eccentric fixation; (b) Fundus appearance: ranging from normal to absent foveal reflex, pigment mottling, narrowing of the retinal vessels, frank atrophy of the retinal pigment epithelium (RPE) in the fovea; (c) VF: small central scotoma; (d) ERG: absent or markedly reduced photopic responses, normal or mildly abnormal scotopic responses; (e) OCT: variable degree of foveal hypoplasia, disruption and/or loss of inner/outer photoreceptor segment junction (IS/OS), now described as EZ band, and an attenuation of RPE layer [14].

### 2.3. Selection of RETplex Genes and Enrichment Procedures

In the RETplex targeted sequencing panel, we included all genes responsible for isolated forms of IRDs that were listed in the RETnet website (http://www.sph.uth.tmc.edu/RetNet/; accessed on 31 March 2014) as well as some genes responsible for both isolated and syndromic forms of IRD. The panel comprised all the coding exons of the above genes as well as the genomic regions covering some previously reported deep intronic mutations [15,16]. Finally, we also included the coding exons of genes and the precursor sequences of microRNAs that, based on our previous results [17,18], can exert a candidate pathogenic role in IRDs. The complete list of genes and sequence elements (*n* = 159) represented in the RETplex panel is reported in Table S1. Capture oligonucleotide probes covering the selected target regions were designed using the HaloPlex^TM^ Target Enrichment System (Agilent Technologies Inc., Santa Clara, CA, USA) according to previously reported protocols [19].

### 2.4. Targeted NGS Analysis

Genomic DNA was extracted from peripheral blood using standard procedures. All procedures were approved by the Ethics Boards of the Università degli Studi della Campania *Luigi Vanvitelli* (Project No. 0006282/2015 approved on 17 March 2015) and adhered to the tenets of the Declaration of Helsinki. All samples were acquired after written informed consent was obtained from the patient or, in the case of children, their legal guardians. DNA quality was assessed and RETplex sequencing libraries were prepared as previously described [20]. Libraries were sequenced using the HiSeq1000 system (Illumina Inc., San Diego, CA, USA). The generated sequences were analyzed using an in-house developed pipeline [19]. Briefly, the generated paired sequencing reads were aligned to the reference genome (UCSC, hg19 build) using the Burrows-Wheeler Alignment (BWA) tool [21] and sorted with SAMtools [22] and Picard (http://picard.sourceforge.net). Genome Analysis Toolkit (GATK) [23] with parameters adapted to the Haloplex-generated sequences was then used to identify insertions-deletions (indel) and single nucleotide variants (SNV). The called SNV and indel variants were annotated using ANNOVAR [24] with: the relative position in genes using the RefSeq87 gene model, amino acid change, presence in dbSNP v137, frequency in the EXAC database (http://exac.broadinstitute.org), 1000 genomes project [25], presence in the Human Gene Mutation Database (HGMD) [26], Clinvar database [27], multiple cross-species conservation [28] and prediction scores of damage on protein activity [29,30,31,32]. The annotated variants were also checked for their presence in an internal variation database, which stores all the variations found in sequencing projects carried out in our Institute. The alignments at candidate positions were visually inspected using the Integrative Genomics Viewer (IGV). All the candidate variants identified were validated by Sanger sequencing.

## 3. Results

### 3.1. Patient Selection

**Figure 1:**
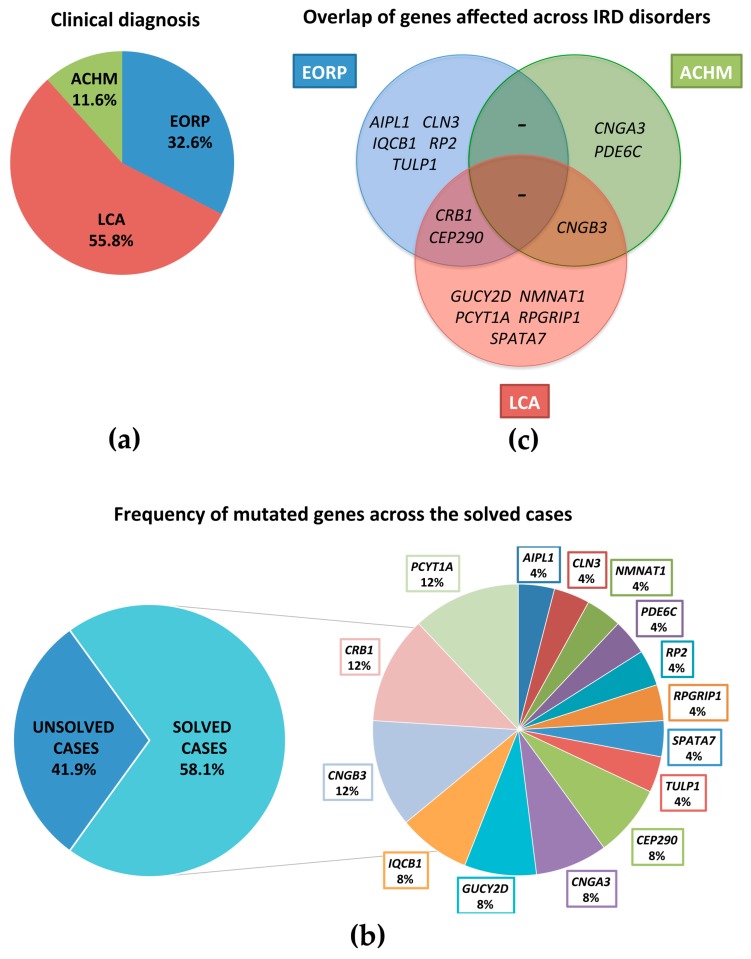
Distribution of clinical diagnosis and mutated genes across the 43 pediatric patients. (**a**) Distribution of clinical diagnosis across the 43 patients with LCA, EORP, and ACHM; (**b**) Frequency of the mutated genes across the solved cases of severe inherited retinal dystrophies (IRD) reported in this study; (**c**) Venn diagram showing the genetic heterogeneity of retinal dystrophies and an overlap between the genetic causes of different IRDs.

We selected for our study a cohort of 43 Italian IRD patients (representative of 41 different families) according to the inclusion criteria mentioned above. Based on a first-level ophthalmological assessment (see Materials and Methods), this cohort was composed of 24 patients affected by LCA (55.8%), 14 by EORP (32.6%), and 5 by ACHM (11.6%) (Figure 1a). All patients were sporadic with the exception of two pairs of sibs.

### 3.2. RETplex Analysis

We set up a targeted NGS-based procedure (RETplex) to identify the genetic basis of disease in IRD patients. In particular, we included in the platform 159 genes, of which 137 with an already demonstrated pathogenic role and 20 genes with a candidate pathogenic role in IRDs (see Table S1). To generate the sequencing libraries, we used the HaloPlex^TM^ Target Enrichment System (Agilent Technologies Inc.) that we have successfully used for other targeted sequencing efforts [19]. We first tested the efficacy of the procedure on a small training subset of three genomic DNAs from IRD patients with already known molecular defects. RETplex allowed us to successfully detect all the previously known mutations in the three patients analyzed.

We then applied the RETplex procedure to the entire cohort of 43 selected patients. In particular, we carried out the analysis on the 41 probands. In the two familial cases, the candidate pathogenic variants were then validated by Sanger sequencing in the affected sib not analyzed by RETplex. This analysis led to the complete identification of the presumably pathogenic variants in 25/43 cases (58.1%) (Table 1). Interestingly, in three additional cases we found single heterozygous mutations in the *CEP290* gene (Table S2). Since the latter variants were all displaying a loss-of-function effect and two of them were previously described as causative [33], we hypothesize that these three patients may harbor a second mutation in *CEP290* that is not detectable using this approach, e.g., a deep intronic mutation or a large copy number variation.

With respect to our first level diagnosis, causative mutations were found in 15 of the 24 LCA, 8 of the 14 EORP, and all 5 ACHM patients. Pathogenic variants were identified in the following genes: *CRB1* in three families (7.0%), *PCYT1A* in two families (3 patients) (7.0%), *CNGB3* in two families (3 patients) (7.0%), *CEP290* in two families (4.7%), *IQCB1* in two families (4.7%), *CNGA3* in two families (4.7%), *GUCY2D* in two families (4.7%), *NMNAT1* in one family (2.3%), *TULP1* in one family (2.3%), *AIPL1* in one family (2.3%), *CLN3* in one family (2.3%), *SPATA7* in one family (2.3%), *RP2* in one family (2.3%), *RPGRIP1* in one family (2.3%), and *PDE6C* in one family (2.3%) (Figure 1b). Twelve of the pathogenic variants identified have never been previously reported (Table 1). Parameters indicative of the pathogenicity of the four novel missense mutations are shown in Table S3.

### 3.3. Clinical Examination

#### 3.3.1. LCA Patients

**Figure 2:**
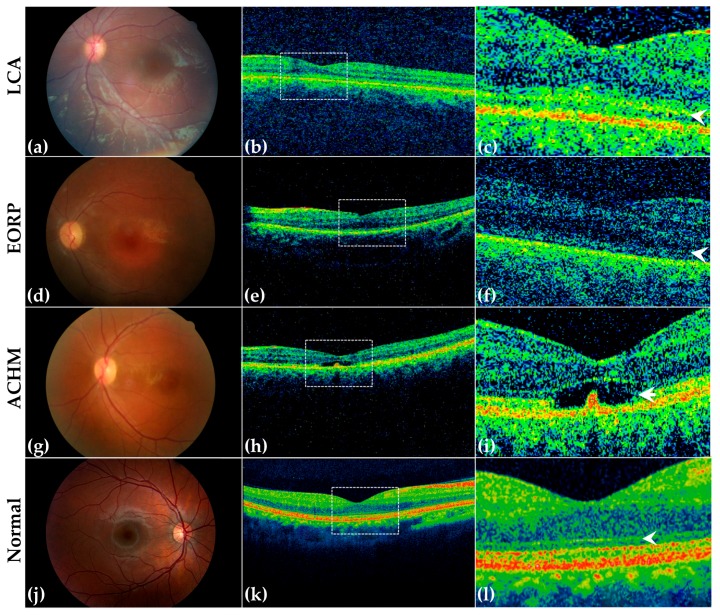
Ophthalmological findings in three representative patients with LCA, EORP, ACHM, and in a normal young proband. Retinography (**a**,**d**,**g**,**j**) and optical coherence tomography (OCT) images (**b**,**e**,**h**,**k**) of representative LCA (pt. 10, mutations in *CNGB3*, **a**–**c**), EORP (pt. 31, mutations in *CEP290*, **d**–**f**) and ACHM (pt. 8, mutations in *CNGB3*, **g**–**i**) patients, and of a normal young proband (**j**–**l**). The retinography and OCT image of a normal retina is also shown (**j**,**k**). Insets (**c**,**f**,**i**,**l**) show a magnified view of the boxed areas in b, e, h and k, respectively. (**a**–**c**) Retinography of the LCA patient shows normal fundus appearance and OCT image demonstrates irregular EZ band (arrowhead in **c**); (**d**–**f**) Fundus image from the EORP patient shows attenuation of the retinal vessels and “salt and pepper” retinal dystrophy. OCT shows absence of the EZ band (arrowhead in **f**) and mild retinal pigment epithelium dystrophy; (**g**–**i**) Retinography and OCT of the ACHM patient reveals pigment mottling and disruption of the EZ band (arrowhead in **i**), respectively. (**j**–**l**) Retinography of a normal young proband and corresponding OCT images. The arrowhead indicates the EZ band (**l**).

The twenty-four patients with LCA had a mean age of 8.2 years (range of 2–17 years). We found that the age of onset ranged from 1 month to 9 months. All patients except one (4%; pt. 5) presented nystagmus. Seven patients (29%) had photophobia (pt. 2, 12, 14, 17, 25, 26, 41) and five patients (21%) showed ocular-digital sign (pt. 12, 23, 25, 26, 38). No cases presented keratoconus or cataract. BCVA in eight cases (33%) could not be evaluated due to the very young age of the patient (pt. 1, 2, 3, 14, 23, 25, 27, 42). BCVA ranged from light perception (LP) to hand motion (HM) in seven patients (29%; pt. 4, 5, 12, 17, 26, 30, 34) and from 20/1000 to 20/200 in nine patients (37.5%; pt. 6, 7, 10, 11, 16, 29, 33, 38, 41). Among the patients in which BCVA testing was performable, the most frequent VA was 20/200 (16%; pt. 10, 16, 29, 33). Refractive errors ranged from −17 D (4) in one patient (4%) to +7.25 D (2) in another patient (4%). Hyperopia was moderate in four patients (16%; pt. 10, 33, 34, 38) and high in four (16%; pt. 1, 2, 16, 25). Myopia was low in one patient (4%; pt. 17) and high in two (8%; pt. 4, 11). Fundus appearance was “salt and pepper” dystrophy in all patients, except three (12%; pt. 10, 25, 42) in which the fundus was normal (Figure 2a) and two (8%; pt. 6, 7; two sisters) in which there were pigmentary deposits resembling “bone spicules”. Because of the patients’ age, ERG was performed only in sixteen patients (66%; pt. 1, 3, 4, 5, 6, 7, 10, 11, 12, 16, 17, 29, 30, 34, 38, 41) in which scotopic and photopic responses were under noise level in twelve (50%; pt. 1, 3, 5, 6, 10, 12, 16, 17, 29, 30, 34, 38). MT in both eyes was between 100 and 328 μm. The ellipsoid zone (EZ) band was absent in eight patients (33%; pt. 2, 6, 7, 12, 16, 17, 29, 34) and irregular in four (16%; pt. 4, 5, 10, 41) (Figure 2b,c, please compare with a normal retina in panels k–l). Among these patients, nine were not found to harbor candidate pathogenic variants in the RETplex analysis (36%). The mutated genes, among LCA patients, were *CEP290*, *CRB1*, *SPATA7*, *GUCY2D*, *NMNAT1*, *CNGB3*, *PCYT1A,* and *RPGRIP1* (Table 1 and Table 2). Clinical and molecular diagnoses were always in agreement except for the patient with mutation in *CNGB3* (Figure 1c). Table 2 summarizes the clinical findings in the cohort of LCA patients.

#### 3.3.2. EORP Patients

The fourteen patients with EORP had a mean age of 10.4 years (2–18 years) with disease onset that ranged from eight months to two years. Six patients (42%) presented nystagmus at 8–9 months (pt. 19, 21, 22, 28, 35, 31). All patients had night-blindness as the earliest symptom. Photophobia was reported in three patients (21.4%; pt. 22, 29, 32). BCVA could not be determined in one case (7.1%; pt. 28) because of the very young age of the patient. In the remaining thirteen cases (92.8%), BCVA ranged from 20/60 to HM (Table 3). Among the patients in which BCVA testing was feasible, the most frequent value was 20/100 (36%; pt. 13, 31, 35, 37, 43). Fundus examination showed “salt and pepper” dystrophy in all patients except one (7.1%; pt. 43) who presented pigmentary deposits resembling bone spicules in mid periphery (Figure 2d). Goldmann visual field examination, which was not always performed owing to the young age of patients, highlighted ring shape scotoma in two patients (14.3%) or tubular visual field in two patients (14.3%). ERG was under noise level in all patients except three (21%; pt. 18, 22, 32) that presented markedly hypovolted photopic traces. MT was between 101 and 247 μm. The EZ band was absent in ten patients (72%; pt. 13, 15, 19, 20, 21, 22, 32, 36, 37, 43) and irregular in three (21.4%; pt. 18, 31, 35) (Figure 2e,f). The mutated genes in the EORP cohort were *CEP290*, *IQCB1*, *TULP1*, *CRB1*, *CLN3*, *AIPL1*, and *RP2* (Table 3). Also in this cohort, overall clinical and molecular diagnosis matched except for the patient 31 bearing *CEP290* mutations (Figure 1c). Table 3 summarizes the clinical findings in the cohort of EORP patients.

#### 3.3.3. ACHM Patients

Finally, the five ACHM cases had a mean age of 12.6 years (range between 10 and 16 years). The onset was around the first year of age. In all patients, color vision was tested by Farnsworth D-15: one patient (20%; pt. 40) had total color vision loss, two (40%; pt. 8, 9) had deutanopia, and the other two (40%; pt. 24, 39) protanopia. Visual acuity (VA) was 20/200 in all patients. Fundus appearance was normal in two patients (40%; pt. 24, 40) and pigment mottling was observed in three patients (60%; pt. 8, 9, 39) (Figure 2g). ERG examination showed that the photopic response was absent or markedly diminished while the scotopic response was normal or mildly abnormal. MT was between 128 and 294 μm. OCT imaging revealed a wide spectrum of photoreceptor integrity, ranging from a continuous ellipsoid zone band at the fovea in two patients (40%; pt. 24, 40) to outer retinal atrophy in three patients (60%; pt. 8, 9, 39) (Figure 2h,i). All of the ACHM patients received a molecular diagnosis. ACHM patients showed *CNGB3*, *CNGA3*, and *PDE6C* mutations. The clinical and molecular diagnoses were always concordant. Table 4 summarizes the clinical findings in the cohort of ACHM patients. One patient refused to perform ERG examination (pt. 8). Patient 8, bearing mutations in the *CNGB3* gene, showed some overlapping clinical features between LCA and ACHM. The disease onset in this patient was at 1 year of age with photophobia and nystagmus. He had deuteranopia at the Farnsworth D-15 test and his BCVA was 20/200. Pigment mottling was observed at fundus examination. However, MT was quite preserved (258 μm in the RE and 294 μm in the LE) and OCT revealed a disruption of the ellipsoid zone band.

## 4. Discussion

To the best of our knowledge, this is the first study that reports an integrated clinical/genetic evaluation in a pediatric IRD cohort and provides an estimation of the frequency of specific gene defects, as well as an analysis of the ellipsoid zone (EZ band) by OCT. A case series of pediatric patients with degenerative retinal disease causing severe visual impairment due to LCA, EORP, and ACHM was recruited. In analyzing these patients, we took into account their different ages (ranging from 2 to 18) that can be relevant for progressive diseases such as IRDs. In our cohort, LCA was the most frequent diagnosis (55.8%) causing severe loss of vision. Following LCA were EORP (32.5%) and ACHM (11.7%). The RETplex success rate was 58.1% (25/43), in line with previous reports [6].

In our LCA patients, the most frequently mutated gene was *CEP290*, when taking into consideration the three cases in which a single heterozygous mutation was found. LCA affects 1 in approximately 30,000 to 80,000 people in the general population, and accounts for more than 5% of all cases of severe visual damage [54]. Mutations in *CEP290* are reported as the most common (20%) cause of LCA [12,55], in agreement with our results.

In EORP, the most frequently mutated gene observed was *IQCB1* (15.4%), which is responsible for syndromic forms of IRDs. In ACHM the most frequently mutated genes were *CNGA3* and *CNGB3* (40%). The ACHM gene distribution in our cohort is in agreement with previous reports [12], while for EORP, our results cannot be compared with other studies because, to the best of our knowledge, this report represents the first example of a detailed analysis of this type of patients.

In this study, we found two notable cases of possible discordancy between clinical and molecular diagnosis. The first one is represented by patient 31 (clinical diagnosis of EORP) who has two putative pathogenic variants (p.R2306*; p.K663*) in the *CEP290* gene, mutations which have been mostly linked to LCA [55]. Previously, *CEP290* mutations in patients with RP had rarely been reported, except for the compound heterozygous mutations (c.4705-1G>T and c.3559delC) in an autosomal recessive RP patient [3] and in another patient with (c.4040G>A and c.3104-2delA) mutation [56]. Patient 31 indeed displays clinical signs highly suggestive of EORP, with preserved visual acuity, absence of oculodigital sign, and abnormal but preserved EZ band [13], which further strengthen the involvement of the *CEP290* gene also in severe forms of RP. The second case of clinical/molecular discordance involves patient 10, who has two frameshift variants in the *CNGB3* gene. So far, *CNGB3* mutations have been reported only in ACHM. Yet, patient 10 has a phenotype compatible with a diagnosis of LCA, in particular considering his ERG, under noise level for both scotopic and photopic response at 1 year of age [12]. Therefore, the present study, for the first time to our knowledge, indicates that mutations in *CNGB3* may also be associated with LCA and not only with ACHM. In light of this finding, it would be advisable to include ACHM genes in LCA-targeted sequencing panels.

The partial discordance between the genotype and the corresponding clinical features found for *CEP290* and for *CNGB3* suggests that additional novel genotype/phenotype correlations can be found for other genes as well. For several patients, the identified gene defect led to a re-evaluation of the patients’ phenotype and the identification of additional abnormalities either not present or too mild to be noted at first evaluation. In fact, patients with mutations in the *IQCB1*, *SPATA7*, *CEP290*, and *CLN3* genes should be re-evaluated also for the systemic involvement correlated to the mutation which is not always detectable at the first observation. For example, the nephronophthisis linked to *IQCB1* mutations could be clinically unmasked belatedly [34,57]. In patient 21, bearing *IQCB1* mutations, a subsequent clinical evaluation led to a diagnosis of polycystic kidney. The latter finding strengthens the need for a careful re-evaluation of the clinical diagnosis following molecular analysis even in cases with apparently isolated forms of IRDs.

We were able to evaluate the macular region by OCT in a notable number of patients, 30 out of 43 (69.8%), despite their young age [58]. In analyzing the macular region, we considered the presence of a relevant MT and of the EZ bands as two important criteria for successful gene therapy [9]. It was possible to evaluate the ellipsoid EZ band in twelve of our LCA patients; in eight of them it was absent, while in four it was irregular. The EORP patients with an irregular ellipsoid EZ band were only three, two with no detected mutations and one with mutations in *CEP290*; in ten EORP patients the EZ band was absent while it was not analyzed in one patient. In our cohort, the patients with ACHM presented the most preserved EZ band (three disruptions and two irregular bands) even if they are the oldest individuals analyzed. It is tempting to speculate that MT and EZ bands are linked to specific genotypes rather than to clinical conditions. Therefore, the evaluation of MT and EZ band is extremely useful, both therapeutically and clinically, to identify a time-frame for effective gene therapy. Our findings suggest that patients with mutations in *CEP290*, *CNGB3*, *CNGA3*, and *PDE6C* genes present fairly well-preserved MT and EZ bands and may benefit more from gene therapy. It must be underlined that when the EZ band is disrupted in the foveolar area and only preserved outside, its reliability as a predictor of the possible improvement in visual acuity may be compromised. This is particularly relevant in the case of ACHM patients. Nevertheless, we believe that our study provides useful information to identify early enough IRD patients that are more likely to reap the benefits of gene therapy. For many conditions that exhibit quick degeneration in combination with functional defects, early gene therapy may be useful to both prevent retinal degeneration and restore visual function. To date, successful proof-of-principle gene therapy data on pre-clinical models are available for most of the genes found to be mutated in the analyzed patients, except for *IQCB1*, *PCYT1A*, *TULP1*, *IMPG1*, and *PDE6C* [55,59,60,61]. Overall, our results clearly indicate that the clinical evaluation of pediatric IRD patients in the absence of a genetic characterization may have limited value in terms of prognostic assessment and of putative therapeutic options.

## 5. Conclusions

This study provides the first detailed clinical and genetic assessment of severe IRDs with infantile onset. We illustrate the distribution of the mutated genes in an Italian IRD pediatric cohort and the importance of novel sequencing technologies to unravel the etiology of IRDs. Genetic results are crucial for a clinical re-evaluation of patients, particularly for children that are often difficult to evaluate clinically because they are unwilling or unable to undergo instrumental testing such as ERG.

## Figures and Tables

**Table 1:** Summary of the genetic findings in the analyzed patients.

Patient ^‡^	Diagnosis	Gene	RefSeq	Allele 1 (nt ^†^)	Allele 1 (prot. ^§^)	Reference	Allele 2 (nt ^†^)	Allele 2 (prot. ^§^)	Reference
**6**	LCA	*PCYT1A*	*NM_005017*	chr3:195966468; c.847C>T	p.(R283*)	[34]	chr3:195975135; c.277G>A	p.(A93T)	[35]
**7**	LCA	*PCYT1A*	*NM_005017*	chr3:195966468; c.847C>T	p.(R283*)	[34]	chr3:195975135; c.277G>A	p.(A93T)	[35]
**8**	ACHM	*CNGB3*	*NM_019098*	chr8:87656009; c.1148delC	p.(T383fs)	[36]	chr8:87660049; c.970A>G	p.(R324G)	this study
**9**	ACHM	*CNGB3*	*NM_019098*	chr8:87656009; c.1148delC	p.(T383fs)	[36]	chr8:87660049; c.970A>G	p.(R324G)	this study
**10**	LCA	*CNGB3*	*NM_019098*	chr8:87656009; c.1148delC	p.(T383fs)	[36]	chr8:87645015; c.1285delT	p.(S429fs)	this study
**12**	LCA	*SPATA7*	*NM_001040428*	chr14:88893049; c.749+1G>A	p.?	[37]	chr14:88903937; c.1115A>G	p.(E372G)	this study
**15**	EORP	*CLN3*	*NM_001042432*	chr16:28497785; c.258_259del	p.(G187fs)	[38]	chr16:28497972; c.161-1G>C	p.?	[38]
**16**	LCA	*CRB*	*NM_001193640*	chr1:197396689; c.1898C>T	p.(T633M)	[39]	chr1:197404419; c.3091delT	p.(C1031fs)	[40]
**19**	EORP	*RP2*	*NM_006915*	chrX:46713166; c.358C>T	p.(R120*)	[41]	-		
**21**	EORP	*IQCB1*	*NM_001023571*	chr3:121491506; c.1066C>T	p.(R356*)	[42]	chr3:121491506; c.1066C>T	p.(R356*)	[42]
**22**	EORP	*AIPL1*	*NM_001033054*	chr17:6329101; c.645G>A	p.(W215*)	[43]	chr17:6329101; c.645G>A	p.(W215*)	[43]
**23**	LCA	*CEP290*	*NM_025114*	chr12:88490755; c.3012delA	p.(K1004fs)	this study	chr12:88477704; c.4732G>T	p.(E1578*)	[44]
**24**	ACHM	*CNGA3*	*NM_001079878*	chr2:99013274; c.1587C>A	p.(F529L)	[45]	chr2:99013274; c.1587C>A	p.(F529L)	[45]
**25**	LCA	*GUCY2D*	*NM_000180*	chr17:7917236; c.2302C>T	p.(R768W)	[46]	chr17:7917236; c.2302C>T	p.(R768W)	[46]
**27**	LCA	*NMNAT1*	*NM_022787*	chr1:10032184; c.53A>G	p.(N18S)	[47]	chr1:10042461; c.542A>G	p.(Y181C)	[48]
**29**	LCA	*PCYT1A*	*NM_005017*	chr3:195966417; c.897+1G>A	p.?	[34]	chr3:195975135; c.277G>A	p.(A93T)	[35]
**31**	EORP	*CEP290*	*NM_025114*	chr12:88449397; c.6916A>T	p.(R2306*)	this study	chr12:88508262; c.1987A>T	p.(K663*)	[49]
**32**	EORP	*IQCB1*	*NM_001023570*	chr3:121527767; c.479_482del	p.(I160fs)	this study	chr3:121515964; c.876+1G>T	p.?	this study
**33**	LCA	*CRB1*	*NM_001193640*	chr1:197237597; c.55_56insT	p.(L19fs)	[50]	chr1:197391051; c.1757G>A	p.(C586Y)	this study
**36**	EORP	*TULP1*	*NM_001289395*	chr6:35467808; c.1286G>A	p.(R429Q)	[51]	chr6:35467808; c.1286G>A	p.(R429Q)	[51]
**37**	EORP	*CRB1*	*NM_001193640*	chr1:197390271; c.977G>A	p.(C326Y)	[52]	chr1:197390271; c.977G>A	p.(C326Y)	[52]
**39**	ACHM	*PDE6C*	*NM_006204*	chr10:95415598; c.2017G>T	p.(D673Y)	this study	chr10:95415598; c.2017G>T	p.(D673Y)	this study
**40**	ACHM	*CNGA3*	*NM_001079878*	chr2:99012747; c.1060C>T	p.(P354S)	[53]	chr2:99012747; c.1060C>T	p.(P354S)	[53]
**41**	LCA	*RPGRIP1*	*NM_020366*	chr14:21762833; c.86-3T>G	p.?	this study	chr14:21793399; c.2225_2226del	p.(G742fs)	this study
**42**	LCA	*GUCY2D*	*NM_000180*	chr17:7912823; c.1669-1G>A	p.?	this study	chr17:7912823; c.1669-1G>A	p.?	this study

LCA: Leber congenital amaurosis; ACHM: achromatopsia; EORP: early onset retinitis pigmentosa; nt: nucleotide; ^‡^ No pathogenic variants were identified in the following LCA (pt. 1, 2, 3, 4, 5, 11, 17, 30, 38) and EORP patients (pt. 13, 18, 20, 28, 35, 43). In the LCA patients 14, 26, and 34 only a single heterozygous mutation was identified in the *CEP290* gene (Table S2). ^†^ Nucleotide variation; ^§^ Predicted protein change.

**Table 2:** Clinical and molecular data of Leber congenital amaurosis patients.

Patient	Age	Age of Onset	Nystagmus	BCVA ^†^ RE/LE ^‡^	Fundus	MT ^§^ (μm) RE/LE ^‡^	EZ Band ^¶^	ERG ^#^ Scotopic RE/LE ^‡^ (μV)	ERG ^#^ Photopic RE/LE ^‡^ (μV)	Mutated Gene
1	2 y	3 m	yes	n.a.	“salt & pepper”	n.a.	n.a.	u.n.l.	u.n.l.	-
2	5 y	6 m	yes	n.a.	“salt & pepper”	229/220	absent	n.a.	n.a.	-
3	4 y	4 m	yes	n.a.	“salt & pepper”	n.a.	n.a.	u.n.l.	u.n.l.	-
4	17 y	6 m	yes	HM/HM	“salt & pepper”	252/119	irregular	15/34.9	53.6/25.0	-
5	14 y	3 m	no	HM/HM	RPE dystrophy	179/181	irregular	u.n.l.	u.n.l.	-
6	18 y	8 m	yes	0.2/0.02	RP	176/120	absent	u.n.l.	u.n.l.	*PCYT1A*
7	10 y	1 m	yes	0.05/0.05	RP	103/103	absent	18.9/25.2	32.6/20.6	*PCYT1A*
10	5 y	4 m	yes	0.1/0.1	normal	168/156	irregular	u.n.l.	u.n.l.	*CNGB3*
11	16 y	9 m	yes	0.05/0.05	RPE dystrophy	n.a.	n.a.	26.2/39.1	27.8/24.28	-
12	6 y	7 m	yes	LP/LP	“salt & pepper”	260/219	absent	u.n.l.	u.n.l.	*SPATA7*
14	9 y	6 m	yes	n.a.	“salt & pepper”	n.a.	n.a.	n.a.	n.a.	*CEP290 ?*
16	5 y	3 m	yes	0.1/0.1	RPE dystrophy	237/328	absent	u.n.l.	u.n.l.	*CRB1*
17	17 y	2 m	yes	HM/HM	“salt & pepper”	149/104	absent	u.n.l.	u.n.l.	-
23	3 y	1 m	yes	n.a.	“salt & pepper”	n.a.	n.a.	n.a.	n.a.	*CEP290*
25	2 y	4 m	yes	n.a.	normal	n.a.	n.a.	n.a.	n.a.	*GUCY2D*
26	8 y	2 m	yes	LP/LP	“salt & pepper”	n.a.	n.a.	n.a.	n.a.	*CEP290 ?*
27	5 y	9 m	yes	n.a.	“salt & pepper”	n.a.	n.a.	n.a.	n.a.	*NMNAT1*
29	3 y	4 m	yes	0.1/0.1	“salt & pepper”	110/100	absent	u.n.l.	u.n.l.	*PCYT1A*
30	11 y	3 m	yes	LP/LP	RPE dystrophy	n.a.	n.a.	u.n.l.	u.n.l.	*-*
33	5 y	3 m	yes	0.1/0.1	“salt & pepper”	n.a.	n.a.	n.a.	n.a.	*CRB1*
34	7 y	1 m	yes	LP/LP	“salt & pepper”	170/146	absent	u.n.l.	u.n.l.	*CEP290 ?*
38	13 y	9 m	yes	0.03/0.02	“salt & pepper”	n.a.	n.a.	u.n.l.	u.n.l.	-
41	8 y	1 m	yes	0.05/0.05	“salt & pepper”	243/242	irregular	u.n.l.	u.n.l.	*RPGRIP1*
42	2 y	1 m	yes	n.a.	normal	n.a.	n.a.	n.a.	n.a.	*GUCY2D*

^†^ Best corrected visual acuity (BCVA); ^‡^ Right eye (RE)/Left eye (LE); ^§^ Macular thickness (MT); ^¶^ Elipsoid zone (EZ) band; ^#^ Electroretinogram (ERG); *?* single heterozygous variant; HM: Hand motion; LP: light perception; m: months; n.a.: not available; RP: Retinitis pigmentosa; RPE: retinal pigment epithelium; u.n.l.: under noise level; y: years.

**Table 3:** Clinical and molecular data of Early Onset Retinitis Pigmentosa patients.

Patient	Age	Age of Onset	Nystagmus	BCVA ^†^ RE/LE ^‡^	Fundus	MT ^§^ (µm) RE/LE ^‡^	EZ Band ^¶^	ERG ^#^ Scotopic RE/LE ^‡^ (μV)	ERG ^#^ Photopic RE/LE ^‡^ (μV)	Mutated Gene
**13**	16 y	2 y	no	0.2/0.2	RPE dystrophy	101/105	absent	u.n.l.	u.n.l.	*-*
**15**	11 y	2 y	no	HM/HM	RPE dystrophy	154/142	absent	n.a.	n.a.	*CLN3*
**18**	16 y	2 y	no	0.3/0.3	“salt & pepper”	205/197	irregular	61.4/69.2	55.5/62.1	*-*
**19**	18 y	8 m	yes	0.1/0.1	RPE dystrophy	114/157	absent	u.n.l.	26.1/13.4	*RP2*
**20**	7 y	2 y	no	0.3/0.3	RPE dystrophy	126/119	absent	u.n.l.	u.n.l.	*-*
**21**	8 y	9 m	yes	0.3/0.3	RPE dystrophy	243/247	absent	u.n.l.	u.n.l.	*IQCB1*
**22**	7 y	9 m	yes	0.008/0.008	“salt & pepper”	129/114	absent	11.6/1.14	4.96/4.66	*AIPL1*
**28**	2 y	8 m	yes	n.a.	normal	n.a.	n.a.	n.a.	n.a.	*-*
**31**	8 y	9 m	yes	0.2/0.2	“salt & pepper”	168/173	irregular	u.n.l.	u.n.l.	*CEP290*
**32**	10 y	2 y	no	0.05/0.05	“salt & pepper”	180/213	absent	14.8/25	12.2/1.22	*IQCB1*
**35**	11 y	9 m	yes	0.2/0.2	normal	137/170	irregular	u.n.l.	u.n.l.	-
**36**	16 y	2 y	no	0.05/0.05	“salt & pepper”	192/215	absent	u.n.l.	u.n.l.	*TULP1*
**37**	5 y	1 y	no	0.2/0.3	RPE dystrophy	101/104	absent	u.n.l.	u.n.l.	*CRB1*
**43**	11 y	8 m	no	0.2/0.2	RP	165/180	absent	u.n.l.	u.n.l.	*-*

^†^ Best corrected visual acuity (BCVA); ^‡^ Right eye (RE)/Left eye (LE); ^§^ Macular thickness (MT); ^¶^ Ellipsoid zone (EZ) band; ^#^ Electroretinogram (ERG); HM: Hand motion; LP: light perception; m: months; n.a.: not available; RP: Retinitis pigmentosa; RPE: retinal pigment epithelium; u.n.l.: under noise level; y: years.

**Table 4:** Clinical and molecular data of Achromatopsia patients.

Patient	Age	Age of Onset	Nystagmus	BCVA ^†^ RE/LE ^‡^	Fundus	MT ^§^ (μm) RE/LE ^‡^	EZ Band ^¶^	ERG ^#^ Scotopic RE/LE ^‡^ (μV)	ERG ^#^ Photopic RE/LE ^‡^ (μV)	Mutated Gene
8	12 y	1 y	yes	0.1/0.1	pigment mottling	258/294	disruption	n.a.	n.a.	*CNGB3*
9	13 y	2 y	yes	0.1/0.1	pigment mottling	166/168	disruption	128/148	7.45/13.1	*CNGB3*
24	10 y	1 y	yes	0.2/0.1	Normal	188/175	irregular	u.n.l.	u.n.l.	*CNGA3*
39	16 y	1 y	yes	0.2/0.2	pigment mottling	148/142	disruption	u.n.l.	u.n.l.	*PDE6C*
40	12 y	1 y	yes	0.2/0.2	Normal	149/128	irregular	61.6/81.9	50.9/63.2	*CNGA3*

^†^ Best corrected visual acuity (BCVA); ^‡^ Right eye (RE)/Left eye (LE); ^¶^ Ellipsoid zone (EZ) band; ^§^ Macular thickness (MT); ^#^ Electroretinogram (ERG); n.a.: not available; u.n.l.: under noise level; y: years.

## Supplementary Materials

The following are available online at www.mdpi.com/2073-4425/8/10/280/s1. Table S1: List of genes and sequences represented in RETplex; Table S2: LCA patients with a single heterozygous variant in the *CEP290* gene; Table S3: In silico characterization of the putative pathogenic missense sequence variants newly-identified in this report.

## References

[1] Huang X.F., Huang F., Wu K.C., Wu J., Chen J., Pang C.P., Lu F., Qu J., Jin Z.B. (2015). Genotype-phenotype correlation and mutation spectrum in a large cohort of patients with inherited retinal dystrophy revealed by next-generation sequencing. Genet. Med..

[2] Zuchner S., Dallman J., Wen R., Beecham G., Naj A., Farooq A., Kohli M.A., Whitehead P.L., Hulme W., Konidari I. (2011). Whole-exome sequencing links a variant in DHDDS to retinitis pigmentosa. Am. J. Hum. Genet..

[3] Neveling K., Collin R.W., Gilissen C., van Huet R.A., Visser L., Kwint M.P., Gijsen S.J., Zonneveld M.N., Wieskamp N., de Ligt J. (2012). Next-generation genetic testing for retinitis pigmentosa. Hum. Mutat..

[4] Audo I., Bujakowska K.M., Leveillard T., Mohand-Said S., Lancelot M.E., Germain A., Antonio A., Michiels C., Saraiva J.P., Letexier M. (2012). Development and application of a next-generation-sequencing (NGS) approach to detect known and novel gene defects underlying retinal diseases. Orphanet J. Rare Dis..

[5] Shanks M.E., Downes S.M., Copley R.R., Lise S., Broxholme J., Hudspith K.A., Kwasniewska A., Davies W.I., Hankins M.W., Packham E.R. (2013). Next-generation sequencing (NGS) as a diagnostic tool for retinal degeneration reveals a much higher detection rate in early-onset disease. Eur. J. Hum. Genet..

[6] Eisenberger T., Neuhaus C., Khan A.O., Decker C., Preising M.N., Friedburg C., Bieg A., Gliem M., Charbel Issa P., Holz F.G. (2013). Increasing the yield in targeted next-generation sequencing by implicating CNV analysis, non-coding exons and the overall variant load: The example of retinal dystrophies. PLoS ONE.

[7] Bennett J., Wellman J., Marshall K.A., McCague S., Ashtari M., DiStefano-Pappas J., Elci O.U., Chung D.C., Sun J., Wright J.F. (2016). Safety and durability of effect of contralateral-eye administration of AAV2 gene therapy in patients with childhood-onset blindness caused by *RPE65* mutations: A follow-on phase 1 trial. Lancet.

[8] Jacobson S.G., Cideciyan A.V., Ratnakaram R., Heon E., Schwartz S.B., Roman A.J., Peden M.C., Aleman T.S., Boye S.L., Sumaroka A. (2012). Gene therapy for leber congenital amaurosis caused by *RPE65* mutations: Safety and efficacy in 15 children and adults followed up to 3 years. Arch. Ophthalmol..

[9] Maguire A.M., Simonelli F., Pierce E.A., Pugh E.N., Mingozzi F., Bennicelli J., Banfi S., Marshall K.A., Testa F., Surace E.M. (2008). Safety and efficacy of gene transfer for Leber's congenital amaurosis. N. Engl. J. Med..

[10] Tao L.W., Wu Z., Guymer R.H., Luu C.D. (2016). Ellipsoid zone on optical coherence tomography: A review. Clin Exp. Ophthalmol..

[11] Marmor M.F., Fulton A.B., Holder G.E., Miyake Y., Brigell M., Bach M. (2009). ISCEV Standard for full-field clinical electroretinography (2008 update). Doc. Ophthalmol. Adv. Ophthalmol..

[12] Chacon-Camacho O.F., Zenteno J.C. (2015). Review and update on the molecular basis of Leber congenital amaurosis. World J. Clin. Cases.

[13] Hamel C. (2006). Retinitis pigmentosa. Orphanet J. Rare Dis..

[14] Kohl S., Jagle H., Wissinger B., Pagon R.A., Adam M.P., Ardinger H.H., Wallace S.E., Amemiya A., Bean L.J.H., Bird T.D., Ledbetter N., Mefford H.C., Smith R.J.H. (1993). Achromatopsia. GeneReviews®.

[15] Den Hollander A.I., Koenekoop R.K., Yzer S., Lopez I., Arends M.L., Voesenek K.E., Zonneveld M.N., Strom T.M., Meitinger T., Brunner H.G. (2006). Mutations in the *CEP290* (*NPHP6*) gene are a frequent cause of Leber congenital amaurosis. Am. J. Hum. Genet..

[16] Webb T.R., Parfitt D.A., Gardner J.C., Martinez A., Bevilacqua D., Davidson A.E., Zito I., Thiselton D.L., Ressa J.H., Apergi M. (2012). Deep intronic mutation in *OFD1*, identified by targeted genomic next-generation sequencing, causes a severe form of X-linked retinitis pigmentosa (RP23). Hum. Mol. Genet..

[17] Alfano G., Conte I., Caramico T., Avellino R., Arno B., Pizzo M.T., Tanimoto N., Beck S.C., Huber G., Dolle P. (2011). *Vax2* regulates retinoic acid distribution and cone opsin expression in the vertebrate eye. Development.

[18] Karali M., Peluso I., Marigo V., Banfi S. (2007). Identification and characterization of microRNAs expressed in the mouse eye. Investig. Ophthalmol. Vis. Sci..

[19] Di Fruscio G., Schulz A., De Cegli R., Savarese M., Mutarelli M., Parenti G., Banfi S., Braulke T., Nigro V., Ballabio A. (2015). Lysoplex: An efficient toolkit to detect DNA sequence variations in the autophagy-lysosomal pathway. Autophagy.

[20] Savarese M., Di Fruscio G., Mutarelli M., Torella A., Magri F., Santorelli F.M., Comi G.P., Bruno C., Nigro V. (2014). MotorPlex provides accurate variant detection across large muscle genes both in single myopathic patients and in pools of DNA samples. Acta Neuropathol. Commun..

[21] Li H., Durbin R. (2009). Fast and accurate short read alignment with Burrows-Wheeler transform. Bioinformatics.

[22] Li H., Handsaker B., Wysoker A., Fennell T., Ruan J., Homer N., Marth G., Abecasis G., Durbin R. (2009). The Sequence Alignment/Map format and SAMtools. Bioinformatics.

[23] DePristo M.A., Banks E., Poplin R., Garimella K.V., Maguire J.R., Hartl C., Philippakis A.A., del Angel G., Rivas M.A., Hanna M. (2011). A framework for variation discovery and genotyping using next-generation DNA sequencing data. Nat. Genet..

[24] Wang K., Li M., Hakonarson H. (2010). ANNOVAR: Functional annotation of genetic variants from high-throughput sequencing data. Nucleic Acids Res..

[25] Abecasis G.R., Altshuler D., Auton A., Brooks L.D., Durbin R.M., Gibbs R.A., Hurles M.E., McVean G.A. (2010). A map of human genome variation from population-scale sequencing. Nature.

[26] Stenson P.D., Mort M., Ball E.V., Shaw K., Phillips A., Cooper D.N. (2014). The Human Gene Mutation Database: Building a comprehensive mutation repository for clinical and molecular genetics, diagnostic testing and personalized genomic medicine. Hum. Genet..

[27] Landrum M.J., Lee J.M., Benson M., Brown G., Chao C., Chitipiralla S., Gu B., Hart J., Hoffman D., Hoover J. (2016). ClinVar: Public archive of interpretations of clinically relevant variants. Nucleic Acids Res..

[28] Goode D.L., Cooper G.M., Schmutz J., Dickson M., Gonzales E., Tsai M., Karra K., Davydov E., Batzoglou S., Myers R.M. (2010). Evolutionary constraint facilitates interpretation of genetic variation in resequenced human genomes. Genome Res..

[29] Adzhubei I., Jordan D.M., Sunyaev S.R. (2013). Predicting functional effect of human missense mutations using PolyPhen-2. Curr. Protoc. Hum. Genet..

[30] Kumar P., Henikoff S., Ng P.C. (2009). Predicting the effects of coding non-synonymous variants on protein function using the SIFT algorithm. Nat. Protoc..

[31] Liu X., Jian X., Boerwinkle E. (2011). dbNSFP: A lightweight database of human nonsynonymous SNPs and their functional predictions. Hum. Mut..

[32] Schwarz J.M., Rodelsperger C., Schuelke M., Seelow D. (2010). MutationTaster evaluates disease-causing potential of sequence alterations. Nat. Methods.

[33] Tory K., Lacoste T., Burglen L., Moriniere V., Boddaert N., Macher M.A., Llanas B., Nivet H., Bensman A., Niaudet P. (2007). High *NPHP1* and *NPHP6* mutation rate in patients with Joubert syndrome and nephronophthisis: Potential epistatic effect of *NPHP6* and *AHI1* mutations in patients with *NPHP1* mutations. J. Am. Soc. Nephrol..

[34] Hoover-Fong J., Sobreira N., Jurgens J., Modaff P., Blout C., Moser A., Kim O.H., Cho T.J., Cho S.Y., Kim S.J. (2014). Mutations in *PCYT1A*, encoding a key regulator of phosphatidylcholine metabolism, cause spondylometaphyseal dysplasia with cone-rod dystrophy. Am. J. Hum. Genet..

[35] Testa F., Filippelli M., Brunetti-Pierri R., Di Fruscio G., Di Iorio V., Pizzo M., Torella A., Barillari M.R., Nigro V., Brunetti-Pierri N. (2017). Mutations in the *PCYT1A* gene are responsible for isolated forms of retinal dystrophy. Eur. J. Hum. Genet..

[36] Sundin O.H., Yang J.M., Li Y., Zhu D., Hurd J.N., Mitchell T.N., Silva E.D., Maumenee I.H. (2000). Genetic basis of total colourblindness among the Pingelapese islanders. Nat. Genet..

[37] Perrault I., Hanein S., Gerard X., Delphin N., Fares-Taie L., Gerber S., Pelletier V., Merce E., Dollfus H., Puech B. (2010). Spectrum of *SPATA7* mutations in Leber congenital amaurosis and delineation of the associated phenotype. Hum. Mutat..

[38] Kousi M., Lehesjoki A.E., Mole S.E. (2012). Update of the mutation spectrum and clinical correlations of over 360 mutations in eight genes that underlie the neuronal ceroid lipofuscinoses. Hum. Mutat..

[39] Den Hollander A.I., ten Brink J.B., de Kok Y.J., van Soest S., van den Born L.I., van Driel M.A., van de Pol D.J., Payne A.M., Bhattacharya S.S., Kellner U. (1999). Mutations in a human homologue of Drosophila crumbs cause retinitis pigmentosa (*RP12*). Nat. Genet..

[40] Den Hollander A.I., Davis J., van der Velde-Visser S.D., Zonneveld M.N., Pierrottet C.O., Koenekoop R.K., Kellner U., van den Born L.I., Heckenlively J.R., Hoyng C.B. (2004). *CRB1* mutation spectrum in inherited retinal dystrophies. Hum. Mutat..

[41] Mears A.J., Gieser L., Yan D., Chen C., Fahrner S., Hiriyanna S., Fujita R., Jacobson S.G., Sieving P.A., Swaroop A. (1999). Protein-truncation mutations in the *RP2* gene in a North American cohort of families with X-linked retinitis pigmentosa. Am. J. Hum. Genet..

[42] Otto E.A., Helou J., Allen S.J., O’Toole J.F., Wise E.L., Ashraf S., Attanasio M., Zhou W., Wolf M.T., Hildebrandt F. (2008). Mutation analysis in nephronophthisis using a combined approach of homozygosity mapping, CEL I endonuclease cleavage, and direct sequencing. Hum. Mutat..

[43] Sohocki M.M., Bowne S.J., Sullivan L.S., Blackshaw S., Cepko C.L., Payne A.M., Bhattacharya S.S., Khaliq S., Qasim Mehdi S., Birch D.G. (2000). Mutations in a new photoreceptor-pineal gene on 17p cause Leber congenital amaurosis. Nat. Genet..

[44] Valente E.M., Silhavy J.L., Brancati F., Barrano G., Krishnaswami S.R., Castori M., Lancaster M.A., Boltshauser E., Boccone L., Al-Gazali L. (2006). Mutations in *CEP290*, which encodes a centrosomal protein, cause pleiotropic forms of Joubert syndrome. Nat. Genet..

[45] Kohl S., Marx T., Giddings I., Jagle H., Jacobson S.G., Apfelstedt-Sylla E., Zrenner E., Sharpe L.T., Wissinger B. (1998). Total colourblindness is caused by mutations in the gene encoding the α-subunit of the cone photoreceptor cGMP-gated cation channel. Nat. Genet..

[46] Lotery A.J., Namperumalsamy P., Jacobson S.G., Weleber R.G., Fishman G.A., Musarella M.A., Hoyt C.S., Heon E., Levin A., Jan J. (2000). Mutation analysis of 3 genes in patients with Leber congenital amaurosis. Arch. Ophthalmol..

[47] Siemiatkowska A.M., van den Born L.I., van Genderen M.M., Bertelsen M., Zobor D., Rohrschneider K., van Huet R.A., Nurohmah S., Klevering B.J., Kohl S. (2014). Novel compound heterozygous *NMNAT1* variants associated with Leber congenital amaurosis. Mol. Vis..

[48] Perrault I., Hanein S., Zanlonghi X., Serre V., Nicouleau M., Defoort-Delhemmes S., Delphin N., Fares-Taie L., Gerber S., Xerri O. (2012). Mutations in *NMNAT1* cause Leber congenital amaurosis with early-onset severe macular and optic atrophy. Nat. Genet..

[49] Halbritter J., Diaz K., Chaki M., Porath J.D., Tarrier B., Fu C., Innis J.L., Allen S.J., Lyons R.H., Stefanidis C.J. (2012). High-throughput mutation analysis in patients with a nephronophthisis-associated ciliopathy applying multiplexed barcoded array-based PCR amplification and next-generation sequencing. J. Med. Genet..

[50] Stone E.M. (2007). Leber congenital amaurosis—A model for efficient genetic testing of heterogeneous disorders: LXIV Edward Jackson Memorial Lecture. Am. J. Ophthalmol..

[51] Ajmal M., Khan M.I., Micheal S., Ahmed W., Shah A., Venselaar H., Bokhari H., Azam A., Waheed N.K., Collin R.W. (2012). Identification of recurrent and novel mutations in *TULP1* in Pakistani families with early-onset retinitis pigmentosa. Mol. Vis..

[52] Simonelli F., Ziviello C., Testa F., Rossi S., Fazzi E., Bianchi P.E., Fossarello M., Signorini S., Bertone C., Galantuomo S. (2007). Clinical and molecular genetics of Leber’s congenital amaurosis: A multicenter study of Italian patients. Investig. Ophthalmol. Vis. Sci..

[53] Wissinger B., Gamer D., Jagle H., Giorda R., Marx T., Mayer S., Tippmann S., Broghammer M., Jurklies B., Rosenberg T. (2001). *CNGA3* mutations in hereditary cone photoreceptor disorders. Am. J. Hum. Genet..

[54] Wang H., Wang X., Zou X., Xu S., Li H., Soens Z.T., Wang K., Li Y., Dong F., Chen R. (2015). Comprehensive Molecular Diagnosis of a Large Chinese Leber Congenital Amaurosis Cohort. Investig. Ophthalmol. Vis. Sci..

[55] Burnight E.R., Wiley L.A., Drack A.V., Braun T.A., Anfinson K.R., Kaalberg E.E., Halder J.A., Affatigato L.M., Mullins R.F., Stone E.M. (2014). *CEP290* gene transfer rescues Leber congenital amaurosis cellular phenotype. Gene Ther..

[56] Shen T., Guan L., Li S., Zhang J., Xiao X., Jiang H., Yang J., Guo X., Wang J., Zhang Q. (2015). Mutation analysis of Leber congenital amaurosis associated genes in patients with retinitis pigmentosa. Mol. Med. Rep..

[57] Estrada-Cuzcano A., Koenekoop R.K., Coppieters F., Kohl S., Lopez I., Collin R.W., De Baere E.B., Roeleveld D., Marek J., Bernd A. (2011). *IQCB1* mutations in patients with leber congenital amaurosis. Investig. Ophthalmol. Vis. Sci..

[58] Alasil T., Keane P.A., Sim D.A., Tufail A., Rauser M.E. (2013). Optical coherence tomography in pediatric ophthalmology: Current roles and future directions. Ophthalmic Surg. Lasers Imaging Retin..

[59] Du W., Tao Y., Deng W.T., Zhu P., Li J., Dai X., Zhang Y., Shi W., Liu X., Chiodo V.A. (2015). Vitreal delivery of AAV vectored Cnga3 restores cone function in *CNGA3^-/-^/Nrl^-/-^* mice, an all-cone model of CNGA3 achromatopsia. Hum. Mol. Genet..

[60] Ku C.A., Pennesi M.E. (2015). Retinal Gene Therapy: Current Progress and Future Prospects. Expert Rev. Ophthalmol..

[61] Michalakis S., Muhlfriedel R., Tanimoto N., Krishnamoorthy V., Koch S., Fischer M.D., Becirovic E., Bai L., Huber G., Beck S.C. (2010). Restoration of cone vision in the *CNGA3*^-/-^ mouse model of congenital complete lack of cone photoreceptor function. Mol. Ther..

